# Upregulation of microRNA-96 and its oncogenic functions by targeting CDKN1A in bladder cancer

**DOI:** 10.1186/s12935-015-0235-8

**Published:** 2015-11-14

**Authors:** Ziyu Wu, Kun Liu, Yunyan Wang, Zongyuan Xu, Junsong Meng, Shuo Gu

**Affiliations:** Department of Urology, Huai’an Hospital Affiliated of Xuzhou Medical College and Huai’an Second People’s Hospital, 62 Huaihai Road South, Huai’an, 223002 People’s Republic of China; Department of Urology, Huai’an First People’s Hospital, Nanjing Medical University, 6 Beijing Road West, Huai’an, 223300 Jiangsu People’s Republic of China

**Keywords:** Bladder cancer, microRNA-96, CDKN1A, Proliferation, Apoptosis

## Abstract

**Background:**

Genome-wide miRNA expression profile has identified microRNA (miR)-96 as one of upregulated miRNAs in clinical bladder cancer (BC) tissues compared to normal bladder tissues. The aim of this study was to confirm the expression pattern of miR-96 in BC tissues and to investigate its involvement in carcinogenesis.

**Methods:**

Quantitative real-time PCR was performed to detect the expression levels of miR-96 in 60 BC and 40 normal control tissues. Bioinformatics prediction combined with luciferase reporter assay were used to verify whether the cyclin-dependent kinase inhibitor CDKN1A was a potential target gene of miR-96. Cell counting kit-8 and apoptosis assays were further performed to evaluate the effects of miR-96-CDKN1A axis on cell proliferation and apoptosis of BC cell lines.

**Results:**

We validated that miR-96 was significantly increased in both human BC tissues and cell lines. According to the data of miRTarBase, CDKN1A might be a candidate target gene of miR-96. In addition, luciferase reporter and Western blot assays respectively demonstrated that miR-96 could bind to the putative seed region in CDKN1A mRNA 3′UTR, and significantly reduce the expression level of CDKN1A protein. Moreover, we found that the inhibition of miR-96 expression remarkably decreased cell proliferation and promoted cell apoptosis of BC cell lines, which was consistent with the findings observed following the introduction of CDKN1A cDNA without 3′UTR restored miR-96.

**Conclusions:**

Our data reveal that miR-96 may function as an onco-miRNA in BC. Upregulation of miR-96 may contribute to aggressive malignancy partly through suppressing CDKN1A protein expression in BC cells.

## Background

Bladder cancer (BC) ranks the 9th most common malignancy worldwide and is one of the costliest to clinically manage [1]. It represents a main cause of morbidity and mortality. Histologically, about 98 % of BC belong to epithelial malignancies, with the vast majority being transitional cell carcinomas (TCC), followed by squamous cell carcinoma (5 %) and adenocarcinoma (2 %) [2]. BC can be divided into two forms: low grade superficial tumors and high grade invasive tumors. Surgical resection is the primary and most effective treatment for patients with low grade superficial tumors [3]. However, there are still no efficient therapeutic strategies for high grade invasive tumors. Although chemotherapy based on BCG may reduce recurrence in BC patients, more than 70 % will recur and approximately 30 % will eventually progress to muscle invasive disease or distant metastasis [4]. Therefore, it is of great significance to identify novel and effective molecular markers for the early-stage diagnosis and for the evaluation of prognosis in patients with BC.

MicroRNAs (miRNAs) are small, endogenous and non-protein-coding RNAs that control gene expression post-transcriptionally by interacting with partially complementary target sites in the 3′-untranslational region (UTR) of specific mRNA targets, either inducing their degradation or impairing their translation [5]. Functionally, miRNAs are implicated in various cellular processes, including cell proliferation, differentiation, cell cycle and apoptosis [6]. Emerging data have shown the abnormal expression of miRNAs may be dramatically associated with several diseases, including cancers [7]. MiRNAs act as either oncogenes or tumor suppressors, and play crucial roles in carcinogenesis, progression, and metastases. The tissue-specific nature of miRNA expression implies that different tumors would express specific miRNA signatures [8]. Especially, previous study of Puerta-Gil et al. identified miR-143, miR-222, and miR-452 as tumor stratification and noninvasive diagnostic biomarkers for BC [9]; Majid et al. [10] reported that miR-23b could function as a tumor suppressor that may confer a proliferative advantage and promote bladder cancer cell migration and invasion; Wu et al. [11] also found that miR-99a could inhibit cell proliferation, migration and invasion by targeting fibroblast growth factor receptor 3 in BC. These findings suggest that it is of critical to identify the targets of miRNAs for understanding the function of miRNAs in cancer development and progression.

Genome-wide miRNA expression profile reported by Yoshino et al. has identified miR-96 as one of upregulated miRNAs in clinical BC tissues compared to normal bladder tissues [12]. Guo et al. [13] identified that miR-96 was downregulated in transitional cell carcinoma tissues compared to normal bladder tissues, and regulated FOXO1-mediated cancer cell apoptosis, while Wang et al. [14] reported that miR-96 was expressed at higher levels in human bladder urothelial carcinomas compared to normal tissues, and found that miR-96 increased invasion and differentiation of human bladder T24 cells and promoted cell growth. These converse findings prompt us to perform this study to confirm the expression pattern of miR-96 in BC tissues and to investigate its involvement in carcinogenesis.

## Results

### Upregulation of miR-96 in BC tissues

**Fig. 1:**
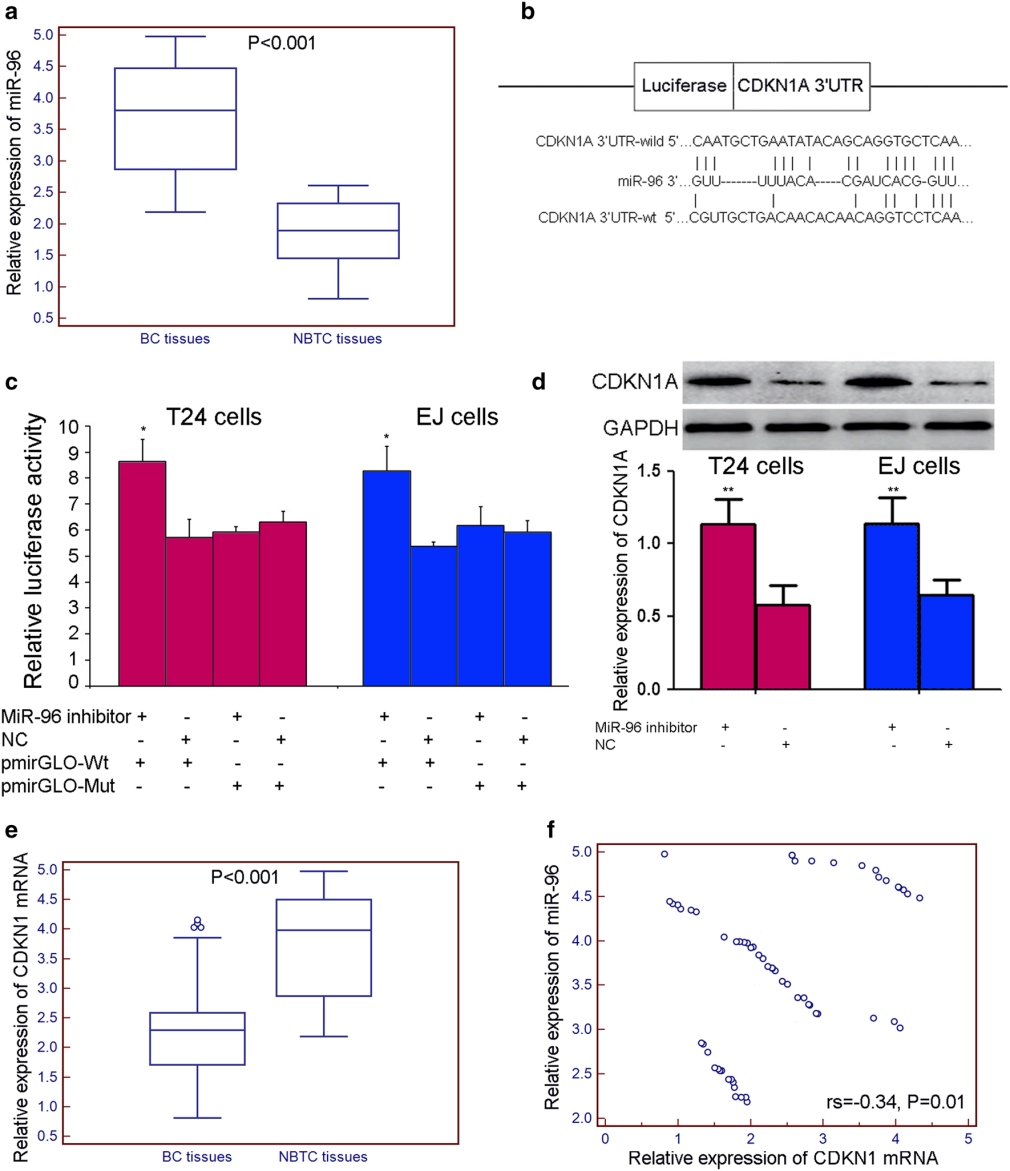
miR-96 expression was upregulated in human bladder cancer (BC) tissues and could negatively regulate the expression of CDKN1A in both BC cells and tissues. **a** Using quantitative real-time PCR, we found that the expression level of miR-96 was significantly increased in BC tissues (n = 60) compared with that in NBTC tissues (n = 40; BC vs. NBTC: 3.69 ± 0.91 vs. 1.86 ± 0.52, P < 0.001); **b** the predicted CDKN1A 3′UTR-wild and CDKN1A 3′UTR-mut binding sequences in miR-96. **c** Co-transfection with miR-96 inhibitor significantly increased the luciferase activity of the reporter containing the wild-type 3′-UTR (*P < 0.05); **d** Western blot analysis also showed that the expression level of CDKN1A protein was significantly up-regulated in two human BC cell lines T24 and EJ transfected with miR-96 inhibitor (**P < 0.001); **e** using quantitative real-time PCR, we found that the expression level of CDKN1A mRNA was significantly decreased in BC tissues (n = 60) compared with that in NBTC tissues (n = 40; BC vs. NBTC: 2.42 ± 1.05 vs. 3.71 ± 0.90, P < 0.001); **f** Spearman correlation analysis showed the negative correlation between miR-96 and CDKNIA mRNA expression levels in human BC tissues (rs = −0.34, P = 0.01)

Using quantitative real-time PCR, we found that the expression level of miR-96 was significantly increased in BC tissues compared with that in NBTC tissues as shown in Fig. 1a (BC vs. NBTC: 3.69 ± 0.91 vs. 1.86 ± 0.52, P < 0.001), offering us an initial evidence that miR-96 might be involved in the development of human BC.

### miR-96 negatively regulates the expression of CDKN1A in BC cells and tissues

We collected the candidate target genes of miR-96 from the experimentally validated microRNA-target interactions database (miRTarBase, http://mirtarbase.mbc.nctu.edu.tw/, Release 4.5), which has accumulated more than 50,000 miRNA–target interactions (MTIs). The data in miRTarBase are collected by manually surveying pertinent literature after data mining of the text systematically to filter research articles related to functional studies of miRNAs [15]. All MTIs in this database are validated experimentally by reporter assay, Western blot, microarray and next-generation sequencing experiments. In the current study, only the MTIs which are validated experimentally by reporter assay, Western blot and quantitative real-time PCR were collected. As a result, a total of seven genes, including FOXO1, FOXO3, HTR1B, Mitf, CDKN1A, PRMT5 and KRAS, have been regarded as candidate target gene of miR-96 as shown in Table 1. According to our literature retrieval, miR-96-FOXO1 axis has been indicated to play a crucial role in bladder carcinogenesis. Li et al. [16] performed Western blotting and dual-luciferase reporter assays to demonstrate that miR-96 could bind to the putative seed region in FOXO3 mRNA 3′UTR, and could significantly decrease the expression of FOXO3 in non-small cell lung cancer cells. Jensen et al. [17] identified an element (A-element) within HTR1B mRNA that confers repression by miR-96. Xu et al. [18] performed target prediction and in vitro functional studies showed that MITF, a transcription factor required for the establishment and maintenance of retinal pigmented epithelium, was a direct target of miR-96. Pal et al. [19] also revealed that the decreased miR-96 expression could augment PRMT5 translation. Recent studies reported that the involvement of miR-96 in colorectal and pancreatic carcinogenesis might be related to its suppression on KRAS expression [20, 21]. In order to validate whether CDKN1A was a potential target of miR-96, luciferase reporter assay was performed. As shown in Fig. 1b, c, co-transfection with miR-96 inhibitor significantly increased the luciferase activity of the reporter containing the wild-type 3′-UTR (P < 0.05). Moreover, Western blot analysis also showed that the expression level of CDKN1A protein was significantly up-regulated in two human BC cell lines T24 and EJ transfected with miR-96 inhibitor (both P < 0.001, Fig. 1d). Importantly, the expression levels of CDKNIA mRNA in BC tissues were dramatically lower than those in NBTC tissues (BC vs. NBTC: 2.42 ± 1.05 vs. 3.71 ± 0.90, P < 0.001, Fig. 1e). The spearman correlation analysis also showed the negative correlation between miR-96 and CDKNIA mRNA expression levels in human BC tissues (rs = −0.34, P = 0.01, Fig. 1f).

**Table 1:** Candidate target genes for miR-96 according to the data of miRTarBase

miRTarBase ID	miRNA	Target gene	Experiments
MIRT001087	hsa-miR-96	FOXO1	QPCR//LRA//WB//IHC//NB
MIRT002447	hsa-miR-96	HTR1B	LRA
MIRT003144	hsa-miR-96	Mitf	IHC//Microarray//qPCR//WB
MIRT003414	hsa-miR-96	CDKN1A	LRA//Microarray//qPCR//WB
MIRT004352	hsa-miR-96	PRMT5	WB
MIRT005450	hsa-miR-96	FOXO3	LRA//qPCR//WB
MIRT005553	hsa-miR-96	KRAS	IHC//NB//WB//LRA

*LRA* luciferase reporter assay, *WB* Western blot, *IHC* immunohistochemistry, *NB* northern blot

Taken together, these data demonstrated that miR-96 could negatively regulate the expression of CDKN1A in both BC cells and tissues.

### Downregulation of miR-96 inhibits cell proliferation of BC cells via regulating CDKN1A mRNA

**Fig. 2:**
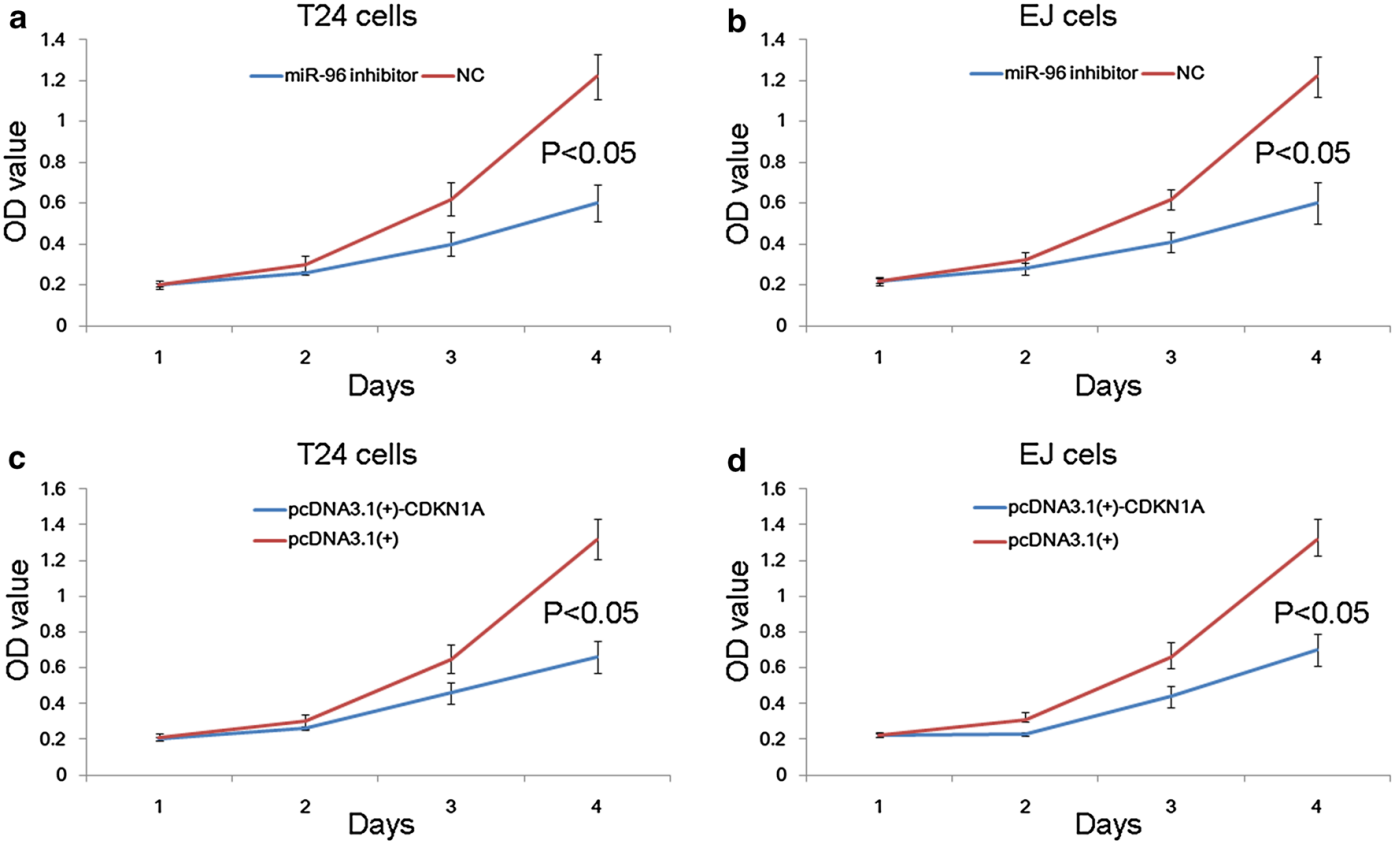
Downregulation of miR-96 inhibits cell proliferation of bladder cancer (BC) cells via regulating CDKN1A mRNA. **a**, **b** Downregulation of miR-96 could remarkably inhibit the growth of both T24 and EJ cells (both P < 0.05). **c**, **d** Transfection with pcDNA3.1(+)-CDKN1A significantly suppressed cell proliferation of both T24 and EJ cells (both P < 0.05)

To determine the effect of miR-96 on cell proliferation of BC cells, T24 and EJ cells were transfected with miR-96 inhibitor and CCK8 assay was performed. As shown in Fig. 2a, b, the downregulation of miR-96 could remarkably inhibit the growth of both T24 and EJ cells (both P < 0.05). After that, recombinant CDKN1A without the 3′UTR sequence (pcDNA3.1(+)-CDKN1A) was transfected into two BC cells. Similar with the observations following the transfection of miR-96 inhibitor, the cell proliferation of both T24 and EJ cells transfected with pcDNA3.1(+)-CDKN1A was significantly suppressed (both P < 0.05, Fig. 2c, d).

### Downregulation of miR-96 promotes apoptosis of BC cells via regulating CDKN1A mRNA

**Fig. 3:**
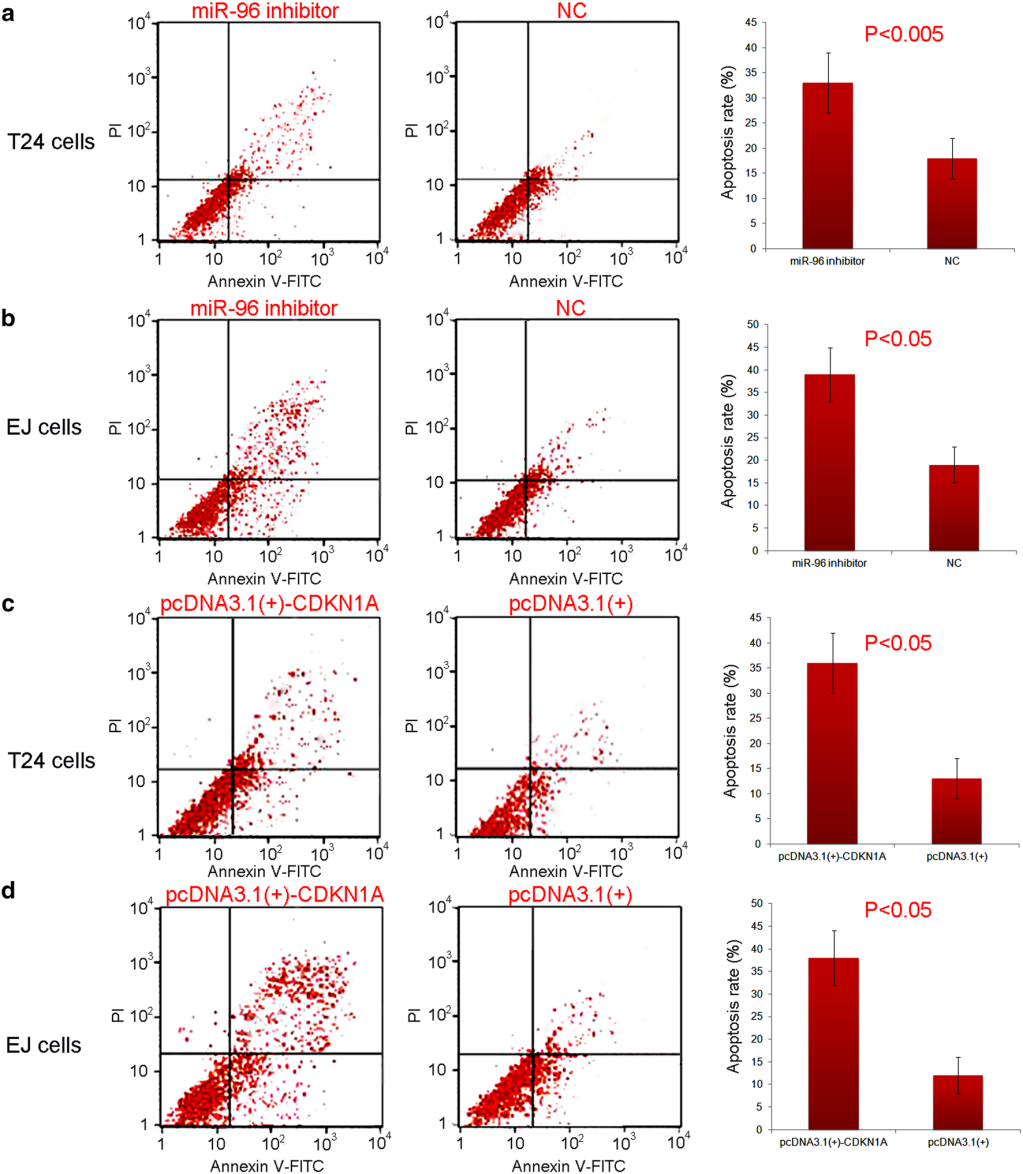
Downregulation of miR-96 promotes apoptosis of bladder cancer (BC) cells via regulating CDKN1A mRNA. **a**, **b** Downregulation of miR-96 could significantly promote apoptosis of both T24 and EJ cells (both P < 0.05); **c**, **d** transfection with pcDNA3.1(+)-CDKN1A significantly promoted apoptosis of both T24 and EJ cells (both P < 0.05)

To determine the effect of miR-96 on cell apoptosis, T24 and EJ cells transfected with miR-96 inhibitor or NC were double-stained with Annexin V-FITC and PI and detected using flow cytometry. As shown in Fig. 3a, b, the downregulation of miR-96 could significantly promote apoptosis of both T24 and EJ cells (both P < 0.05), which was consistent with the observation after the transfection of the recombinant CDKN1A without the 3′UTR sequence (pcDNA3.1(+)-CDKN1A) (both P < 0.05, Fig. 3c, d).

## Discussion

Growing evidence show a direct link between miRNAs and human cancers. miRNAs often play a role in the control of carcinogenesis, cell proliferation and apoptosis of malignant cells by regulating the expression of the corresponding target genes. In the current study, we confirmed the upregulation of miR-96 in human BC tissues, which was in line with the previous genome-wide miRNA expression profile reported by Yoshino et al. [12]. In addition, bioinformatics prediction, combined with luciferase reporter and Western blot assays, identified CDKN1A as a potential target of miR-96, which could negative regulate the expression level of CDKN1A in both BC cells and tissues. Moreover, we found that the inhibition of miR-96 expression remarkably decreased cell proliferation and promoted cell apoptosis of BC cell lines, consisting with the findings observed following the introduction of CDKN1A cDNA without 3′UTR restored miR-96.

MiR-96, together with miR-182 and miR-183, belongs to the miR-183 family, which is located proximally on human chromosome 7, a region containing several oncogenes and commonly amplified in cancers [17]. MiR-96, miR-182 and miR-183 share the same transcription start site (chr7: 129207158), suggesting that these miRNAs may be coordinately expressed and function together during carcinogenesis [18]. Recent studies showed that miR-96 was frequently increased in several human cancers. MiR-96 has been reported to exert an oncogenic effect in non-small cell lung cancer, esophageal cancer, breast cancer, hepatocellular carcinoma, and prostate cancer, respectively by inhibiting the expression of FOXO3, RECK, FOXO3a and FOXO1 [16, 20–23]. On the other hand, miR-96 has been reported to function as a tumor suppressor in several cancers. For instance, miR-96 directly targeted the GTPase Kras (KRAS) oncogene in pancreatic cancer cells and ectopic expression of miR-96 through a synthetic miRNA precursor inhibited KRAS, dampened Akt signaling, and triggered apoptosis in cells [24, 25]. Regarding to BC, the conflict results on the expression patterns of miR-96 in human BC tissues have been reported by different research groups [13, 14]. Liu et al. [26] also indicated that miR-183/96/182 cluster might play oncogenic roles in BC. Similarly, we here found that the upregulation of miR-96 could enhance cellular proliferation and inhibit apoptosis of BC cells, implying that miR-96 might contribute to the carcinogenesis and aggressive progression of BC.

In addition, we validated that CDKN1A (p21), which is an important inhibitor of the cell-cycle, regulator of the DNA damage response and effector of the tumor suppressor p53 [27], was a potential target gene of miR-96 in BC cells. As a major player in mammalian cell cycle progression, CDKN1A inhibits cyclin/cdk2 complexes and plays a crucial role in mediating growth arrest when cells are exposed to DNA damaging agents, implying its tumor suppressive role [28]. Several factors, including Ap2, BRCA1, C/EBPa, E2F-1/E2F-3, Sp1/Sp3, Smads and STAT, activate the transcription of CDKN1A [29]. Moreover, CDKN1A is also involved in terminal differentiation, replicative senescence and protection from p53-dependent and -independent apoptosis [30]. Cazier et al. [31] performed the whole-genome sequencing of BC revealed somatic CDKN1A mutations and clinicopathological associations with mutation burden. Liu et al. [32] also found that TP53/CDKN1A double-mutant BC cells had a unique dependence on Chk1 activity for the G2-M cell-cycle checkpoint in response to chemotherapy-induced DNA damage. CDKN1A could be regulated by several miRNAs in many cancers, including miR-519d, miR-375, miR-31 and miR-663 [33–35]. In the current study, we found that miR-96 negatively regulated CDKN1A expression, and downregulation of miR-96 inhibited cell proliferation and promoted apoptosis in BC cells by targeting CDKN1A.

In conclusion, our data reveal that miR-96 may function as an onco-miRNA in BC. Upregulation of miR-96 may contribute to aggressive malignancy partly through suppressing CDKN1A protein expression in BC cells.

## Methods

### Ethics, consent and permissions

This work was approved by the Research Ethics Committee of Huai’an Hospital Affiliated of Xuzhou Medical College, Huai’an Second People’s Hospital and Huai’an First People’s Hospital, Nanjing Medical University. Written informed consent was obtained from all of the patients. All specimens were handled and made anonymous according to the ethical and legal standards.

### Consent to publish

We have obtained consent to publish from the participant (or legal parent or guardian for children) to report individual patient data.

### Patients and tissue samples

Sixty TCC samples included 40 non muscle-invasive TCC samples which was collected by transurethral resection of bladder tumor and 20 muscle-invasive TCC tissue which was collected by radical cystectomy. Histological identification of TCC was evaluated based on the World Health Organization criteria. The clinicopathological features of 60 patients with TCC were summarized in Table 2. In addition, 40 age-matched patients undergoing suprapubic transvesical prostatectomy, lithocystotomy and cystostomy for non-malignant diseases were collected for NBTC controls.

**Table 2:** Clinicopathological features of 60 patients with bladder cancer

Clinicopathological features	Case	Percent (%)
Age (years)		
≤50	40	66.7
>50	20	33.3
Gender		
Male	45	75.0
Female	15	25.0
Growth pattern		
Papillary	40	66.7
Non-papillary	20	33.3
Pathological stage		
pTa	28	46.7
pT1	15	25.0
pT2	10	16.7
pT3	5	8.3
pT4	2	3.3
Tumor grade		
G1	15	25.0
G2	35	58.3
G3	10	16.7
Lymph node metastasis		
Negative	40	66.7
Positive	20	33.3

### Cell culture

Two human bladder cancer cell lines, T24 and EJ, were purchased from the Type Culture Collection of the Chinese Academy of Sciences (Shanghai, China) and cultured in RPMI-1640 medium supplemented with 10 % fetal bovine serum (FBS) (Gibco, Grand Island, NY, USA) under a humidified air atmosphere of 5 % CO_2_ at 37 °C.

### Cell transfection

A specific miR-96-5p inhibitor (sequence: 5′-AGC AAA AAU GUG CUA GUG CCA AA-3′, Shanghai GenePharma Co. Ltd, Shanghai, China) and a negative control (NC, sequence: 5′-CAG UAC UUU UGU GUA GUA CAA-3′, Shanghai GenePharma Co. Ltd, Shanghai, China) were commercially purchased. Two human BC cell lines T24 and EJ were both plated at a density of 1.8 × 10^5^ cells/well in six-well plates. Transient transfection was conducted using Lipofectamine™ 2000 (Invitrogen, Carlsbad, CA, USA) following the manufacturer’s instructions which the cells reached ~60 % confluence. Forty-eight hours after transfection, the cells were harvested for further experiments.

### RNA extraction and quantitative real-time PCR

To detect the expression levels of miR-96 and CDKN1 mRNA in TCC and NBTC tissues, and two human BC cell lines, total RNAs were extracted by Trizol reagent (Invitrogen, Carlsbad, CA, USA), and 1 μg of RNA was reversely transcripted. For miR-96, quantitative real-time PCR was performed using a high-specificity miR-96 Detection Kit (Stratagene Corp., La Jolla, CA, USA) in conjunction with an ABI 7500 thermal cycler, according to the manufacturer’s instruction. For CDKN1 mRNA, the PCR primer was: 5′-CAG AGG AGG CGC CAA GAC AG-3′ (forward) and 5′-CCT GAC GGC GGA AAA CGC-3′ (reverse). The RT-PCR kit (BD Biosciences, NJ, USA) was used in conjunction with an ABI 7500 thermal cycler following the manufacturer’s protocol. U6 and GAPDH were used internal controls for the expression of miR-96 and CDKN1 mRNA, respectively. Relative miR-96 and CDKN1 mRNA expression levels were calculated using the comparative threshold cycle (Ct) method [36].

### Western blot analysis

To detect the expression levels of CDKN1A protein in human BC cell lines T24 and EJ transfected with miR-96 inhibitor or NC, the Western blot analysis was performed according to the previous studies [37, 38]. In brief, total proteins of transfected cells were extracted using RIPA buffer containing phenylmethanesulfonyl fluoride (PMSF) and were quantified by a BCA kit (Thermo, Waltham, MA, USA). Proteins were separated by sodium dodecyl sulfate polyacrylamide gel electrophoresis (SDS-PAGE) and transferred to polyvinylidene difluoride (PVDF) membranes. The membranes were incubated overnight at 4 °C with diluted primary antibodies (mouse anti-human CDKN1A antibody, 1:100, Cat. #AM2134b, Abgent Inc., San Diego, CA, USA; or mouse anti-human GAPDH, 1:100, Cat. #AM1020b, Abgent Inc., San Diego, CA, USA). Following extensive washing with Tris-buffered saline containing Tween 20 (TBST), the secondary antibodies were incubated at room temperature for 1 h (HRP labeled goat anti-mouse IgG, 1:1000, Sangon Biotech, Shanghai, China) for 1 h, and the membranes were detected by ECL system (Pierce, Rockford, IL, USA).

### Luciferase reporter assay

To demonstrate whether CDKN1A mRNA was a target of miR-96, the luciferase reporter assay was performed according to the previous studies [37, 38]. In brief, the human CDKN1A mRNA fragments containing putative seed regions for miR-96 were amplified by PCR using the following primers: sense 5′-TTC TTC TAG AGG AAG CCC TAA TCC-3′ and antisense 5′-TCC CTT CTA GAA AGA TCT ACT CCC C-3′. The PCR product was inserted into psiCHECK2 luciferase miRNA expression reporter vector (Promega, Madison, WI, USA). Mutation experiment was performed using a fast mutation kit (NEB, Ipswich, Canada). Two human BC cell lines T24 and EJ were cultured in 24-well plate for 24 h and co-transfected with 150 nM of miR-96 inhibitor or NC and WT or Mut 3′-UTR of CDKN1A using Lipofectamine 2000. After that, transfected cells were collected, and the relative luciferase activity was assayed using Dual-Luciferase Reporter Assay System (Promega, Wisconsin, WI, USA). The results were normalized with Renilla luciferase. Each reporter plasmid was transfected at least three times (on different days) and each sample was assayed in triplicate.

### Cell counting kit-8 assay

Cell counting kit-8 (CCK8) assay was performed to observe the cell proliferation of human BC cell lines T24 and EJ transfected with miR-96 inhibitor and NC using CCK8 solution (Dojindo, Gaithersburg, MD, USA) according to the manufacturer’s protocol. Optical density was measured at 450 nm using a microplate reader.

### Apoptosis assay

Apoptosis assay was performed to observe the cell apoptosis of human BC cell lines T24 and EJ transfected with miR-96 inhibitor and NC using Annexin V-FITC Apoptosis Detection Kit I (BestBio, Shanghai, China) according to the manufacturer’s protocol. The cells were analyzed using flow cytometry (FACSCalibur, Beckman Coulter, Miami, FL, USA).

### Statistical analysis

Statistical analysis in the current study was performed by the software of SPSS version 13.0 for Windows (SPSS Inc, IL, USA). All the experiments were done in triplicate and continuous variables were expressed as Mean ± SD. Student’s t test and one-way analysis of variance (ANOVA) were used to determine the statistical significance of differences, which were considered statistically significant when *p* was less than 0.05.
